# Applicability of cCR assessment criteria in pMMR rectal cancer patients treated with neoadjuvant chemoradiotherapy combined with immunotherapy

**DOI:** 10.1097/JS9.0000000000002795

**Published:** 2025-06-23

**Authors:** Junpeng Pei, Haijun Xu, Qiushi Dong, Yuye Gao, Yonglin Huang, Xingyu Xie, Lei Huang, Boyang Qu, Yongjiu Chen, Shouxin Yang, Aiwen Wu

**Affiliations:** a State Key Laboratory of Holistic Integrative Management of Gastrointestinal Cancers, Beijing Key Laboratory of Carcinogenesis and Translational Research, Unit III, Gastrointestinal Cancer Center, Peking University Cancer Hospital & Institute, Beijing, China; b Key Laboratory of Carcinogenesis and Translational Research (Ministry of Education/Beijing), Department of Radiology, Peking University Cancer Hospital & Institute, Beijing, China

**Keywords:** complete response, endoscopy, immunotherapy, locally advanced rectal cancer, magnetic resonance imaging, neoadjuvant chemoradiotherapy

## Abstract

**Background:**

This study aimed to assess whether clinical complete response (cCR) criteria developed for neoadjuvant chemoradiotherapy (NACRT) are applicable to patients with proficient mismatch repair locally advanced rectal cancer (LARC) who are treated with NACRT combined with immunotherapy (NAICRT).

**Methods:**

This retrospective study included 49 LARC patients who received NAICRT and 128 who received NACRT. Clinical response was assessed using two established criteria: the Memorial Sloan Kettering Cancer Center (MSKCC) criteria and the Chinese Watch-and-Wait Database (CWWD) criteria. These criteria integrate findings from digital rectal examination, endoscopy, MRI, and, optionally, carcinoembryonic antigen levels and biopsy results. Pathological complete response (pCR), determined from surgical specimens, was used as the reference standard. The diagnostic performance of these criteria in predicting pCR was evaluated using sensitivity, specificity, accuracy, and area under the curve (AUC).

**Results:**

Among the 177 patients, 54 achieved pCR (18 in the NAICRT group and 36 in the NACRT group). MSKCC and CWWD criteria showed comparable performance in the NACRT and NAICRT groups, respectively. Sensitivities were 0.11 and 0.28; specificities, 0.99 and 1.00; accuracies, 0.74 and 0.73; and AUCs, 0.55 and 0.64, with no significant differences between groups. Endoscopy demonstrated sensitivities of 0.28 and 0.61, specificities of 0.96 and 0.90, accuracies of 0.77 and 0.80, and AUCs of 0.67 and 0.76 in the NACRT and NAICRT groups, respectively, with a significant difference in sensitivity (*P* = 0.02). MRI showed sensitivities of 0.50 and 0.39, specificities of 0.91 and 0.90, accuracies of 0.80 and 0.71, and AUCs of 0.71 and 0.65, without significant differences.

**Conclusions:**

The cCR assessment criteria originally developed for NACRT are also applicable to NAICRT patients, showing excellent specificity but suboptimal sensitivity, which may lead to overtreatment of patients with actual pCR. Future studies should focus on enhancing sensitivity to better support organ preservation.

**HIGHLIGHTS**

- The cCR assessment criteria developed for NACRT are also applicable to NAICRT patients, achieving a specificity of 100%.
- The current cCR assessment criteria exhibit a high false-negative rate in both the NAICRT and NACRT groups.
- Emerging technologies such as ctDNA analysis, advanced imaging, and artificial intelligence should be explored to improve post-treatment tumor response evaluation.

## Introduction

Locally advanced rectal cancer (LARC) presents significant clinical challenges due to its high risk of local recurrence and distant metastasis^[1]^. Achieving pathological complete response (pCR) following neoadjuvant treatment is closely associated with improved long-term survival, making it a crucial therapeutic goal^[2]^. Accordingly, the current standard of care involves neoadjuvant chemoradiotherapy (NACRT) followed by total mesorectal excision (TME) and postoperative chemotherapy^[3]^, achieving pCR in approximately 15–30% of cases and yielding favorable prognoses for complete responders^[4-7]^. For patients who achieve clinical complete response (cCR) after neoadjuvant therapy, the Watch-and-Wait (W&W) strategy has emerged as an alternative to surgery^[8]^, aiming to preserve organ function, avoid perioperative complications, and improve quality of life without compromising oncological safety^[8,9]^. In line with the TITAN 2025 guideline on AI transparency reporting^[10]^, we confirm that no artificial intelligence tools were used in the conduct or reporting of this study.

In recent years, immune checkpoint inhibitors (ICIs) have shown high efficacy in mismatch repair-deficient (dMMR) or microsatellite instability-high (MSI-H) rectal cancers^[11-13]^. However, the majority of rectal cancers – approximately 85–90% – are mismatch repair-proficient (pMMR) or microsatellite stable (MSS), for which ICI monotherapy has proven largely ineffective^[14]^. To address this limitation, combination strategies incorporating ICIs and conventional therapies have been explored. Notably, neoadjuvant chemoradiotherapy combined with immunotherapy (NAICRT) has shown encouraging results, with reported pCR rates up to 56.5%, offering new potential for organ preservation in pMMR/MSS patients^[15-21]^.

Despite these advances, a major clinical challenge lies in accurately assessing cCR after NAICRT. Traditional cCR evaluation criteria based on digital rectal examination (DRE), endoscopy, magnetic resonance imaging (MRI), and carcinoembryonic antigen (CEA) levels were developed for NACRT^[22-24]^ and may not account for immune-related changes seen in tumors treated with NAICRT. In dMMR/MSI-H tumors, immunotherapy can induce histological alterations such as lymphocyte infiltration, necrosis, fibrosis, and edema, potentially resulting in pseudo-progression and false-positive imaging findings^[25-27]^. Whether similar phenomena occur in pMMR/MSS rectal cancer after NAICRT remains unclear.

This study primarily aims to evaluate the applicability of traditional cCR criteria, developed for NACRT, in assessing treatment response in pMMR/MSS rectal cancer patients following NAICRT. In addition, we compared the diagnostic performance of two commonly used criteria, the Memorial Sloan Kettering Cancer Center (MSKCC) criteria and the Chinese Watch-and-Wait Database (CWWD) criteria. Clarifying these issues will help refine surveillance strategies, guide treatment decisions, and ultimately improve outcomes for this large subgroup of rectal cancer patients.

## Materials and methods

### Patients and data collection

**Figure 1.**
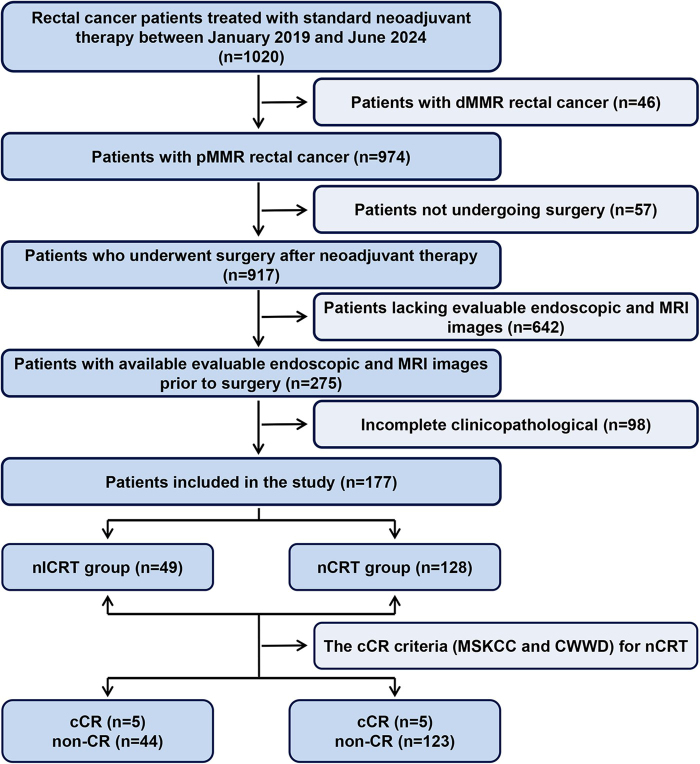
Screening flow diagram. Abbreviations: dMMR, deficient mismatch repair; pMMR, proficient mismatch repair; MRI, magnetic resonance imaging; NAICRT, neoadjuvant chemoradiotherapy plus immunotherapy; NACRT, neoadjuvant chemoradiotherapy.

This retrospective study analyzed medical records, endoscopic findings, and MRI scans of rectal cancer patients who received NACRT or NAICRT followed by surgical resection at our hospital between January 2019 and October 2024. The inclusion criteria were as follows: (1) histologically confirmed adenocarcinoma; (2) pMMR/MSS tumors; (3) clinical staging of T3-T4 or any lymph node-positive (N+); (4) no evidence of distant metastasis based on radiological examinations; (5) treatment involving radiotherapy as part of the neoadjuvant regimen; (6) administration of neoadjuvant chemotherapy or chemotherapy combined with ICI; (7) evaluation after neoadjuvant therapy using endoscopy and pelvic MRI. All patients were staged for clinical and pathological tumor classification according to the 8th edition of the American Joint Committee on Cancer (AJCC) staging system^[28]^. The detailed patient screening process is depicted in Fig. 1. Ethical approval for this study was obtained from the Institutional Review Board of the hospital. The work has been reported in line with the STARD criteria^[29]^.

### Neoadjuvant therapy and surgery

In the long-course radiotherapy regimen, the primary tumor and high-risk areas received a total radiation dose of 41.8–50.4 Gray (Gy), with fractionated doses of 1.8–2.0 Gy per session over 22–28 sessions. During radiotherapy, patients took oral capecitabine at a dose of 825 mg/m^2^ twice daily, 5 days a week. Chemotherapy typically consisted of the capecitabine and oxaliplatin (CapeOx) regimen, consisting of oxaliplatin (130 mg/m^2^ intravenously) and capecitabine (1000 mg/m^2^ orally twice daily for 14 consecutive days). Immunotherapy involved agents such as PD-1 inhibitors (camrelizumab, toripalimab, tislelizumab, sintilimab), a PD-L1 inhibitor (atezolizumab), or a TIGIT inhibitor (tiragolumab). Immunotherapy regimens included monotherapy or dual therapy, such as the combination of atezolizumab and tiragolumab. After neoadjuvant therapy, all patients underwent surgical treatment, including low anterior resection (LAR), abdominoperineal resection (APR), or local excision. Lymph node data were not available for patients who underwent local excision, as these procedures do not include lymph node dissection.

### Endoscopy

**Figure 2.**
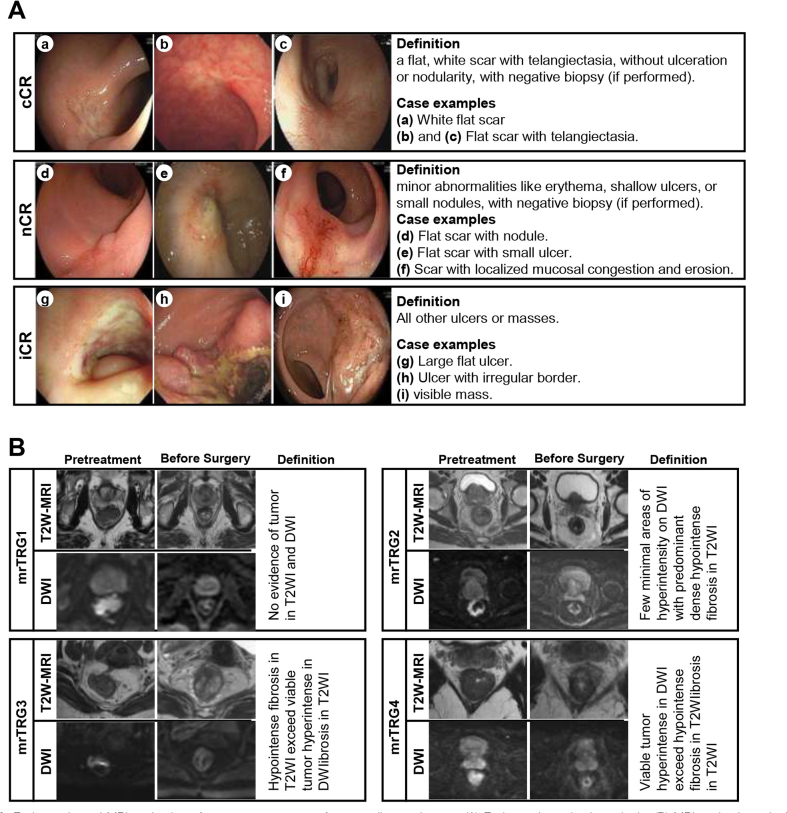
Endoscopic and MRI evaluation of treatment response after neoadjuvant therapy. (A) Endoscopic evaluation criteria. (B) MRI evaluation criteria. Abbreviations: ccR, complete clinical response; nCR, near-complete response; iCR, incomplete clinical response; MRI: magnetic resonance imaging; T2W-MRI, T2-weighted magnetic resonance imaging; DWI, diffusion-weighted imaging.

After neoadjuvant therapy, each patient underwent at least one endoscopic examination, with the final preoperative results evaluated. Tumor regression was classified into five types based on endoscopic images and reports:(1) flat scars; (2) flat scars with nodules, shallow ulcers, or mild mucosal abnormalities; (3) large flat ulcers; (4) ulcers with irregular or raised edges; and (5) tumorous masses. Biopsies were performed when endoscopic findings were inconclusive. Positive results indicate residual tumor or high-grade dysplasia. Negative results, however, could not definitively indicate complete response (CR) due to potential sampling errors. According to predefined criteria, CR was characterized by a flat, white scar with telangiectasia, without ulceration or nodularity, and negative biopsy results (if performed). Near-complete response (nCR) was defined as irregular mucosa with small mucosal nodules or minor mucosal abnormalities, superficial ulceration, and mild persisting erythema of the scar, with negative biopsy results (if performed). Incomplete response (iCR) referred to the presence of a visible tumor. Three experienced clinicians independently and consistently evaluated the anonymized endoscopic findings. The specific image examples of the endoscopic assessment criteria are shown in Fig. 2.

### Imaging

MRI was conducted at baseline and post-treatment to assess the primary tumor and regional lymph nodes. Suspicious lymph nodes were defined by a short axis >10 mm or >5 mm with malignant features such as irregular borders or intensity heterogeneity. Experienced radiologists reviewed T2-weighted (T2W) and diffusion-weighted MRI (DWI) images (b1000 and apparent diffusion coefficient (ADC)) blinded to endoscopic or pathological data. Tumor regression was graded using the MRI-based magnetic resonance tumor regression grading (mrTRG) system: mrTRG1 for complete response (CR), mrTRG2 for near-complete response (nCR), and mrTRG3–5 for iCR^[30]^. The specific image examples of the MRI assessment criteria are shown in Fig. 2, with mrTRG5 not displayed in the figure.

**Figure 3.**
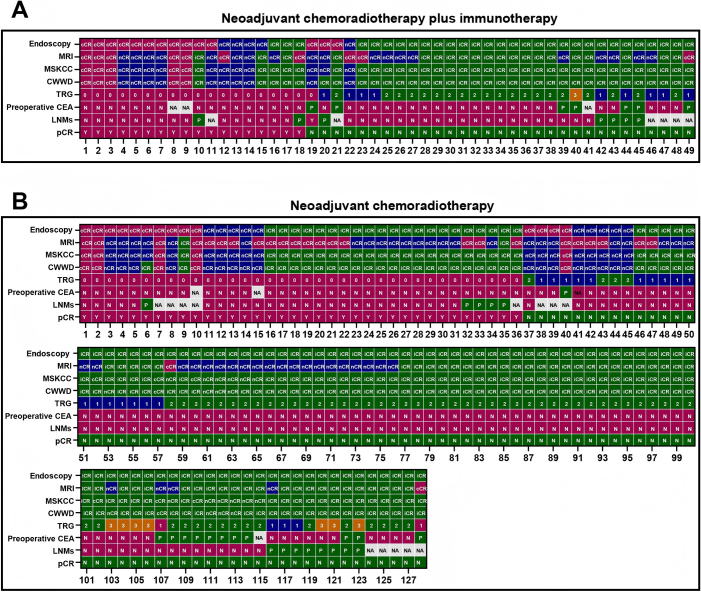
Assessment of response in each rectal cancer patient after neoadjuvant therapy using different evaluation methods. (A) Neoadjuvant chemoradiotherapy with immunotherapy (NAICRT) group. (B) Neoadjuvant chemoradiotherapy (NACRT) group. Abbreviations: dMMR, deficient mismatch repair; pMMR, proficient mismatch repair; MRI, magnetic resonance imaging; NAICRT, neoadjuvant chemoradiotherapy plus immunotherapy; NACRT, neoadjuvant chemoradiotherapy.

### Clinical assessment

cCR was assessed based on two guidelines: the MSKCC criteria (see Table S1, available at: http://links.lww.com/JS9/E441)^[24]^ and the 2024 CWWD consensus (see Table S2, available at: http://links.lww.com/JS9/E441)^[22]^. The CWWD criteria additionally incorporated optional CEA testing and replaced subjective imaging descriptions from the MSKCC criteria with the standardized MRI tumor regression grade (mrTRG) system. Accordingly, cCR under the CWWD consensus is defined as: (1) normal DRE, (2) endoscopic findings consistent with cCR, (3) mrTRG1, and (4) negative CEA (if performed). In clinical practice, DRE is usually performed together with endoscopy, and due to the frequent incompleteness of DRE records in retrospective data, it was not analyzed separately in this study.

### Pathological evaluation

The reference standard was the histopathological analysis of surgical specimens by experienced gastrointestinal pathologists. The results were standardized to include TRG, tumor stage, and lymph node status. pCR was defined as the absence of tumor cells in the primary tumor and lymph nodes (ypT0N0) after curative surgery or the absence of tumor cells in the lesion (ypT0) after local excision.

### Statistical analysis

Baseline demographic, clinical, and treatment characteristics were summarized using descriptive statistics. Categorical variables were expressed as frequencies (percentages), while continuous variables were presented as medians (range) or means ± standard deviation. Comparisons of continuous variables were performed using the Wilcoxon rank-sum test, and comparisons of categorical variables were conducted using the chi-square test or Fisher’s exact test. The predictive performance of endoscopy, MRI, and MSKCC was evaluated using the area under the curve (AUC), accuracy, sensitivity, and specificity. A *P* value of ≤0.05 was considered statistically significant in all analyses. All statistical analyses were conducted using SPSS version 22.0 (IBM Corp., Armonk, NY, USA) and R version 4.4.1 (R Foundation for Statistical Computing, Vienna, Austria).

## Results

### Patient characteristics

A total of 177 patients were included in the study, of whom 128 received NACRT and 49 received NAICRT. There were no significant differences between the two groups in terms of demographic and clinical characteristics, including gender, age, clinical stage before treatment, and baseline biomarker levels (Table 1). In terms of pCR, 28.1% (38/128) of patients in the NACRT group achieved pCR, while 36.7% (18/49) achieved pCR in the NAICRT group (Table 1). The detailed baseline characteristics of the patients are shown in Table 1, and the patient selection process is illustrated in Fig. 1. The response assessment of each rectal cancer patient after neoadjuvant treatment is shown in Fig. 3.

**Table 1:** Clinical characteristics

Variable	NACRT n (%)	NAICRT n (%)	*P* value
Pretreatment			
Total, n	128	49	
Age,Mean ± SD,y	57.8 ± 10.7	56.5 ± 10.4	0.43
Gender			0.86
Male	91 (71.31)	34 (69.4)	
Female	37 (28.9)	15 (30.6)	
cT stage			0.59
cT2	10 (7.8)	2 (4.1)	
cT3	91 (71.1)	39 (79.6)	
cT4	27 (21.1)	8 (16.3)	
cN stage			0.68
cN0	5 (3.9)	1 (2.0)	
cN1	33 (25.8)	10 (20.4)	
cN2	90 (70.3)	38 (77.6)	
CEA level			0.22
Negative (<5 ng/mL)	75 (58.6)	31 (63.3)	
Positive (≥5 ng/mL)	45 (35.2)	18 (36.7)	
NA	8 (6.3)	0 (0.0)	
CA19-9 level			0.56
Negative (<35 ng/mL)	102 (79.7)	42 (85.7)	
Positive (≥35 ng/mL)	14 (10.9)	5 (10.2)	
NA	12 (9.4)	2 (4.1)	
Before Surgery			
Endoscopic findings			0.03
Flat scar with or without neovascularization	14 (10.9)	14 (28.6)	
Flat scar with nodule, shallow ulcer, or mild mucosal abnormality	16 (12.5)	5 (10.2)	
Large flat ulcer	20 (15.6)	10 (20.4)	
Irregular or raised ulcer	55 (43.0)	15 (30.6)	
Visible mass	23 (18.9)	5 (10.2)	
Endoscopic response			<0.01
cCR	14 (10.9)	14 (28.6)	
nCR	10 (7.8)	5 (10.2)	
iCR	104 (81.3)	30 (61.2)	
mrTRG			1.00
mrTRG1	26 (20.3)	10 (20.4)	
mrTRG2	48 (37.5)	19 (38.8)	
mrTRG3	53 (41.4)	20 (40.8)	
mrTRG4	1 (0.8)	0 (0.0)	
CWWD			0.12
cCR	5 (3.9)	5 (10.2)	
nCR	17 (13.3)	10 (20.4)	
iCR	106 (85.2)	34 (69.4)	
MSKCC			0.09
cCR	5 (3.9)	5 (10.2)	
nCR	18 (14.1)	11 (22.4)	
iCR	105 (82.0)	33 (67.3)	
TRG			0.50
TRG0	38 (29.7)	19 (38.8)	
TRG1	20 (15.6)	9 (18.4)	
TRG2	63 (49.2)	20 (40.8)	
TRG3	7 (5.5)	1 (2.0)	
pCR			0.28
YES	36 (28.1)	18 (36.7)	
NO	92 (71.9)	31 (63.3)	
pT stage			0.07
pT0	38 (29.7)	19 (38.8)	
pT1	4 (3.1)	3 (6.1)	
pT2	52 (40.6)	10 (20.6)	
pT3	33 (25.8)	17 (34.7)	
pT4	1 (0.8)	0	
pN stage			0.21
pN0	110 (85.9)	38 (77.6)	
pN1	13 (10.2)	9 (18.4)	
pN2	3 (2.3)	0	
NA	2^*^ (1.6)	2^*^ (4.1)	
Number of positive lymph nodes, median (range)	0 (0–5)	0 (0–2)	0.33
Number of harvested lymph nodes, median (range)	10 (0–38)	11 (2–26)	0.45
CEA level			0.67
Negative (<5 ng/mL)	102 (79.7)	36 (73.5)	
Positive (≥5 ng/mL)	13 (10.2)	7 (14.3)	
NA	13 (10.2)	6 (12.2)	
CA19-9 level			0.66
Negative (<35 ng/mL)	102 (79.7)	38 (77.6)	
Positive (≥35 ng/mL)	6 (4.7)	1 (2.0)	
NA	20 (15.6)	10 (20.4)	
Surgical type			0.62
TME	125 (97.7)	47 (95.9)	
LE	3 (2.3)	2 (4.1)	
The time interval between endoscopy and MRI examination, median (IQR), d	17 (8–30)	18 (11–27)	0.96
Time from treatment initiation to surgery, median (IQR), d	157 (120–197)	160 (126–215)	0.37
Time from the end of radiotherapy to endoscopy, median (IQR), d	88 (71–123)	98 (80–125)	0.79
Time from the end of radiotherapy to MRI, median (IQR), d	64 (55–108)	79 (63–107)	0.71
Time from the end of radiotherapy to surgery, median (IQR), d	105 (82–147)	105 (86–139)	0.43

cCR, clinical complete response; iCR, incomplete clinical response; IQR, interquartile range; d, day; LE, local excision; MRI, magnetic resonance imaging; n, number; NA, not available; nCR, near-complete response; NACRT, neoadjuvant chemoradiotherapy; NAICRT, neoadjuvant chemoradiotherapy combined with immunotherapy; pCR, pathological complete response; pN, pathological node stage; pT, pathological tumor stage; SD, standard deviation; TME, total mesorectal excision; *, Lymph node data were not available for patients who underwent local excision, as these procedures do not include lymph node dissection.

### Tumor morphological features

We compared the tumor morphological features between the NACRT and NAICRT groups. A higher proportion of patients in the NAICRT group exhibited flat scars, with or without neovascularization (28.6% vs. 10.9%), suggesting a higher likelihood of cCR. For other features, such as flat scars accompanied by nodules, shallow ulcers, or mild mucosal abnormalities, the response rates were comparable between the two groups. However, the NACRT group showed higher proportions of irregular or raised ulcers and visible masses (43.0% vs. 30.6% and 18.9% vs. 10.2%, respectively), suggesting poorer tumor response in this group.

### Results from different assessment methods

In the NACRT group, the proportions of patients identified as having cCR based on endoscopy, MRI, MSKCC, and CWWD criteria were 10.9% (14/128), 20.3% (26/128), 3.9% (5/128), and 3.9% (5/128), respectively. In the NAICRT group, the proportions were 28.6% (14/49), 20.4% (10/49), 10.2% (5/49), and 10.2% (5/49). The concordance with pCR was 71.4% (10/14), 69.2% (18/26), 80.0% (4/5), and 80.0% (4/5) in the NACRT group, and 78.6% (11/14), 70.0% (7/10), 100% (5/5), and 100.0% (5/5) in the NAICRT group (Table 2).

**Table 2:** Comparative analysis of treatment responses in rectal cancer patients undergoing NACRT and NAICRT

	NACRT (n = 128)		NAICRT (n = 49)	
	pCR n (%)	Non-pCR n (%)	pCR n (%)	Non-pCR n (%)
Total, n	36 (28.1)	102 (71.9)	18 (36.7)	31 (63.3)
Endoscopic findings				
Flat scar with or without neovascularization	10 (71.4)	4 (28.6)	11 (78.6)	3 (21.4)
Flat scar with nodule, shallow ulcer, or mild mucosal abnormality	7 (43.8)	9 (56.3)	4 (80.0)	1 (20.0)
Large flat ulcer	6 (30.0)	14 (70.0)	0 (0.0)	10 (100)
Irregular or raised ulcer	9 (13.8)	46 (86.2)	2 (13.3)	13 (86.7)
Visible mass	4 (17.4)	19 (82.6)	1 (20.0)	4 (80.0)
Endoscopic response				
cCR	10 (71.4)	4 (28.6)	11 (78.6)	3 (21.4)
Non-cCR	26 (22.8)	88 (77.2)	7 (20.0)	28 (80.0)
nCR	5 (50.0)	5 (50.0)	4 (80.0)	1 (20.0)
iCR	21 (19.4)	83 (80.6)	3 (10.0)	27 (90.0)
MRI				
cCR	18 (69.2)	8 (30.8)	7 (70.0)	3 (30.0)
Non-cCR	18 (17.6)	84 (82.4)	11 (28.2)	28 (71.8)
nCR	16 (33.3)	32 (66.7)	8 (42.1)	11 (57.9)
iCR	2 (3.7)	52 (96.3)	3 (15.0)	17 (75.0)
MSKCC				
cCR	4 (80.0)	1 (20.0)	5 (100.0)	0 (0.0)
Non-cCR	32 (26.0)	91 (74.0)	13 (29.5)	31 (70.5)
nCR	10 (55.6)	8 (44.4)	8 (72.7)	3 (27.3)
iCR	22 (21.0)	83 (79.0)	5 (15.2)	28 (84.8)
CWWD				
cCR	4 (80.0)	1 (20.0)	5 (100.0)	0 (0.0)
Non-cCR	32 (26.0)	91 (74.0)	13 (29.5)	31 (70.5)
nCR	9 (52.9)	8(47.1)	8 (80.0)	2 (20.0)
iCR	23 (21.7)	83 (78.3)	5 (14.7)	29 (85.3)

cCR, clinical complete response; CWWD, the Chinese Watch & Wait Database Research Cooperation Group; iCR, incomplete clinical response; nCR, near-complete response; MRI, magnetic resonance imaging; MSKCC, Memorial Sloan Kettering Cancer Center; n, number; NACRT, neoadjuvant chemoradiotherapy; NAICRT, neoadjuvant chemoradiotherapy combined with immunotherapy; pCR, pathological complete response.

**Table 3:** Comparison of diagnostic performance between NACRT and NAICRT in rectal cancer

Parameter		Endoscopic	MRI	MSKCC & CWWD
Sensitivity (95% CI)	NACRT	0.28 (0.14–0.45)	0.50 (0.33–0.67)	0.11 (0.03–0.26)
	NAICRT	0.61 (0.36–0.83)	0.39 (0.17–0.64)	0.28 (0.1–0.53)
	*P*	0.02	0.45	0.18
Specificity (95% CI)	NACRT	0.96 (0.89–0.99)	0.91 (0.84–0.96)	0.99 (0.94–1.00)
	NAICRT	0.90 (0.74–0.98)	0.90 (0.74–0.98)	1.00 (0.89–1.00)
	*P*	0.36	0.87	0.32
Accuracy (95% CI)	NACRT	0.77 (0.68–0.84)	0.80 (0.72–0.86)	0.74 (0.66–0.82)
	NAICRT	0.80 (0.66–0.90)	0.71 (0.57–0.83)	0.73 (0.59–0.85)
	*P*	0.67	0.24	0.92
AUC (95% CI)	NACRT	0.62 (0.54–0.69)	0.71 (0.62–0.79)	0.55 (0.50–0.60)
	NAICRT	0.76 (0.63–0.88)	0.65 (0.52–0.77)	0.64 (0.53–0.75)
	*P*	0.07	0.46	0.15

CWWD, the Chinese Watch & Wait Database Research Cooperation Group; AUC, the area under the curve; MRI, magnetic resonance imaging; MSKCC, Memorial Sloan Kettering Cancer Center; n, number; NACRT, neoadjuvant chemoradiotherapy; NAICRT, neoadjuvant chemoradiotherapy combined with immunotherapy.

### Diagnostic efficacy

The diagnostic performance of different assessment methods is summarized in Table 3. The MSKCC and CWWD criteria demonstrated comparable diagnostic performance for evaluating cCR. Both criteria demonstrated better performance in the NAICRT group, with sensitivity and AUC being higher than in the NACRT group, although these differences were not statistically significant. Specificity and accuracy remained high for both groups.

Endoscopic assessment demonstrated significantly higher sensitivity in the NAICRT group (0.61 vs. 0.28, *P* = 0.02). Although the difference in AUC between the groups was greater in the NAICRT group, it was not statistically significant (0.76 vs. 0.62, *P* = 0.07). MRI evaluation showed lower sensitivity and AUC in the NAICRT group compared to the NACRT group, but these differences were not statistically significant. Specificity remained consistent between the two groups.

A more detailed description of the results is provided in the Supplementary Materials.

## Discussion

Organ preservation is crucial for rectal cancer patients, and the use of ICIs has significantly increased pCR rates, facilitating the implementation of the W&W strategy^[15,17-19,21,31]^. Therefore, accurately assessing the response to neoadjuvant therapy is essential. Our findings suggest that the cCR assessment criteria developed for NACRT can also be effectively applied to patients receiving NAICRT, with the addition of immunotherapy not inducing distinct response patterns in pMMR/MSS patients.

The MSKCC and CWWD criteria were used to evaluate cCR^[22,24]^. Although the NAICRT group demonstrated higher sensitivity and AUC compared to the NACRT group, these differences were not statistically significant, and both criteria yielded consistent results. Notably, the CWWD criteria, which incorporate biopsy results and CEA levels (if applicable), improved the detection rate of pCR among nCR patients, thereby enhancing the safety of implementing the “watch-and-wait” strategy for nCR patients. Specifically, endoscopic evaluation revealed significantly higher sensitivity in the NAICRT group compared to the NACRT group (*P* = 0.02), although the difference in AUC was not statistically significant. In contrast, MRI evaluation showed lower sensitivity in the NAICRT group compared to the NACRT group, but again, the difference was not statistically significant. These findings support the applicability of current cCR criteria across both treatment modalities, with potentially improved performance following NAICRT.

In contrast, the findings of Zhai *et al*^[32]^ differ from ours, as they reported that the sensitivity and accuracy of clinical response assessment were lower in the NAICRT group than in the NACRT group. Several key factors may explain this discrepancy. First, in Zhai *et al*’s study, the NAICRT group received short-course radiotherapy (SCRT), while the NACRT group received long-course radiotherapy (LCRT). This inconsistency in treatment regimens limits the comparability and reliability of their results. Second, our NAICRT group differed from that of Zhai *et al* – our patients received long-course radiotherapy (LCRT), while theirs received short-course radiotherapy (SCRT). Different fractionation schemes can lead to distinct immune changes in the tumor microenvironment^[15,33]^. LCRT may allow better TME remodeling and longer immune activation, improving response assessment accuracy and contributing to the differences in clinical outcomes. Finally, although standardized evaluation criteria exist, subjective factors, such as interpretation of endoscopic findings and MRI results, may still influence the evaluation process and impact the consistency of results.

This study further evaluated the morphological characteristics of the tumors observed through endoscopy after treatment in both groups. Compared to the NACRT group, the NAICRT group showed a greater tendency for tumors to regress into flat scars with or without capillary dilation (i.e., cCR) after treatment, and there was a higher consistency with pCR among these patients. Several studies have shown that radiation can cause tumor necrosis, disrupt membrane integrity, and release damage-associated molecular patterns, which leads to the exposure of antigens from primary tumor cells and triggers immunogenic cell death^[34]^. Furthermore, radiotherapy stimulates an inflammatory response within the TME, involving the production, recruitment, activation, and release of various cytokines, chemokines, and immune cells^[35-37]^. Therefore, the synergy between radiotherapy and ICIs enhances immune surveillance and the ability to eliminate tumor cells, thereby promoting significant tumor shrinkage or regression and reducing the occurrence of residual tumors. Although the proportion of flat scars with nodules, shallow ulcers, or mild mucosal abnormalities (i.e., nCR) is similar between the NAICRT and NACRT groups (10.2% vs. 12.5%), a higher percentage of patients in the NAICRT group achieved pCR post-surgery (80.0% vs. 43.8%). This may be due to immunotherapy accelerating tumor clearance, resulting in more thorough tumor eradication at the molecular level, which in turn creates differences in the tumor’s appearance and the clearance process. Pseudo-progression refers to clinical tumor progression confirmed as a treatment response on pathology^[38]^. Our study found no pseudo-progression in pMMR rectal cancer patients after NAICRT, indicating that immunotherapy did not trigger specific responses in pMMR patients, unlike in dMMR patients^[26,39]^.

Endoscopy assesses tumor residuals in the mucosal layer, while MRI evaluates the tumor in the muscular layer. MRI results show no significant differences between the groups. Despite a higher pCR rate in the NAICRT group (36.7% vs. 28.1%), MRI did not detect a higher cCR rate (20.4% vs. 20.3%), suggesting lower MRI sensitivity in this group. Sensitivity analysis (0.39 vs. 0.50) further supports this, though the difference is not statistically significant. Reduced MRI sensitivity may be due to changes induced by immunotherapy, such as inflammation, fibrosis, and necrosis, which can lead to signal abnormalities and misinterpretation as tumor residue^[25,40]^.

The insufficient sensitivity in cCR assessment, leading to a high false-negative rate, remains a major clinical challenge for patients undergoing neoadjuvant therapy. This limitation may result in some patients with favorable treatment responses missing the opportunity for watch-and-wait strategies and organ preservation. Notably, the addition of immunotherapy has improved the sensitivity of both endoscopic and overall assessments, indicating that current criteria remain applicable in combined treatment regimens and offer enhanced detection capability. This improvement facilitates more accurate identification of patients who have achieved pCR, supporting the rational application of W&W strategies and contributing to better organ preservation and quality of life. However, current cCR definitions – primarily based on DRE, endoscopy, and MRI – remain limited by interobserver variability, lack of molecular-level insight, and insufficient predictive power. These traditional tools often rely on morphological changes, which may not fully reflect underlying tumor biology. As a result, even with improved systemic treatments such as NAICRT, a significant proportion of patients with pathological complete response still undergo radical surgery due to false-negative assessments. To address these limitations, future studies should aim to refine cCR definitions by integrating more objective and functional biomarkers^[41-50]^. Promising strategies include dynamic monitoring of circulating tumor DNA (ctDNA), which can reflect minimal residual disease at a molecular level^[41,42]^; application of functional imaging such as PET/MRI or FAPI-based imaging to assess tumor metabolism and fibrosis^[45]^; and the development of artificial intelligence (AI)-based predictive models using radiomics, endoscopy, and pathology data^[48-50]^. These approaches may improve both sensitivity and specificity, ultimately enabling more accurate identification of patients suitable for non-operative management. Future research should also explore the long-term oncologic safety of W&W strategies in patients selected using enhanced assessment tools and evaluate whether the integration of novel modalities can safely expand the criteria for organ preservation.

This study has several limitations. As a single-center, retrospective analysis, there is potential for selection bias, and the relatively small sample size limits the statistical power to detect differences in key outcomes. Moreover, the current sample size may not be sufficient to detect small-to-moderate differences in diagnostic performance metrics such as AUC, further limiting the robustness of subgroup comparisons. Future research should address these limitations by exploring prospective multicenter trials to overcome potential biases associated with single-center studies. Enrolling a larger and more representative patient cohort would improve statistical power and enhance the generalizability of the findings. Additionally, incorporating advanced imaging techniques and other novel biomarkers, such as ctDNA, could help address the current challenges in evaluating cCR and improving patient management. Moreover, it would be valuable to investigate the impact of different immunotherapy regimens on treatment response patterns and to assess the long-term outcomes associated with these innovative approaches.

In summary, this study demonstrates that traditional cCR assessment criteria remain applicable and clinically informative for pMMR rectal cancer patients treated with NAICRT, when a standardized long-course radiotherapy protocol is used. Our findings reduce methodological heterogeneity seen in previous studies, clarify inconsistent results, and highlight that the integration of immunotherapy does not necessitate abandoning established evaluation frameworks. Rather, these criteria can be further refined through emerging tools such as molecular markers and AI-based models, supporting more accurate, individualized decision-making in the immunotherapy era.

## Conclusions

In conclusion, the cCR assessment criteria originally developed for NACRT are also applicable to NAICRT patients, showing excellent specificity but limited sensitivity. This insufficient sensitivity may lead to overtreatment, as some patients who achieve pCR may still undergo unnecessary surgery. To address this, future research should focus on enhancing the sensitivity of cCR assessment by integrating ctDNA monitoring, functional imaging, and AI-based tools to improve patient selection for organ preservation.

## Data Availability

The datasets used and/or analyzed during the current study are available from the corresponding author upon reasonable request.

## Research registration unique identifying number (UIN)

This study was registered on ClinicalTrials (NCT06794099).
